# Choosing the optimal dose in sublingual immunotherapy: Rationale for the 300 index of reactivity dose

**DOI:** 10.1186/s13601-015-0088-1

**Published:** 2015-12-23

**Authors:** Pascal Demoly, Gianni Passalacqua, Moises A. Calderon, Tarik Yalaoui

**Affiliations:** Allergy Division, Pulmonology Department, Hôpital Arnaud de Villeneuve, University Hospital of Montpellier, Montpellier, France; Allergy and Respiratory Diseases, IRCCS San Martino-IST, University of Genoa, Genoa, Italy; Section of Allergy and Clinical Immunology, Imperial College London-NHLI, Royal Brompton Hospital, London, UK; Global Medical Affairs Department, Stallergenes, Antony, France

**Keywords:** 300 index of reactivity, Adherence, Allergen-specific immunotherapy, Dose-ranging, Grass pollen allergy, House dust mite allergy, Sublingual immunotherapy

## Abstract

Sublingual immunotherapy (SLIT) is an effective and well-tolerated method of treating allergic respiratory diseases associated with seasonal and perennial allergens. In contrast to the subcutaneous route, SLIT requires a much greater amount of antigen to achieve a clinical effect. Many studies have shown that SLIT involves a dose–response relationship, and therefore it is important to use a proven clinically effective dose from the onset of treatment, because low doses are ineffective and very high doses may increase the risk of side effects. A well-defined standardization of allergen content is also crucial to ensure consistent quality, potency and appropriate immunomodulatory action of the SLIT product. Several methods of measuring antigenicity are used by manufacturers of SLIT products, including the index of reactivity (IR), standardized quality tablet unit, and bioequivalent allergy unit. A large body of evidence has established the 300 IR dose of SLIT as offering optimal efficacy and tolerability for allergic rhinitis due to grass and birch pollen and HDM, and HDM-induced moderate, persistent allergic asthma. The 300 IR dose also offers consistency of dosing across a variety of different allergens, and is associated with higher rates of adherence and patient satisfaction. Studies in patients with grass pollen allergies showed that the 300 IR dose has a rapid onset of action, is effective in both adults and children in the short term and, when administered pre-coseasonally in the long term, and maintains the clinical benefit, even after cessation of treatment. In patients with HDM-associated AR and/or asthma, the 300 IR dose also demonstrated significant improvements in symptoms and quality of life, and significantly decreased use of symptomatic medication. The 300 IR dose is well tolerated, with adverse events generally being of mild or moderate severity, declining in frequency and severity over time and in the subsequent courses. We discuss herein the most important factors that affect the selection of the optimal dose of SLIT with natural allergens, and review the rationale and evidence supporting the use of the 300 IR dose.

## Background

Allergic rhinitis (AR) is one of the most common chronic conditions worldwide, affecting an estimated 500 million people [1]. The recommended approach to the management of AR is a combination of patient education (with specific allergen avoidance when feasible), symptomatic pharmacotherapy and allergen immunotherapy (AIT) [2–4]. The goals of AR treatment are short-term symptomatic relief, improvement in quality of life (QoL), and of the modification of the immune response of the allergic disease [5].

Allergen immunotherapy is based on the repeated administration of extracts of the symptom-eliciting allergens, with the aim of reducing the clinical and immunological response to these allergens and, ultimately, inducing a persistent immunological tolerance [6, 7]. AIT is effective in improving symptoms, reducing the use of symptomatic drugs, and is the only disease-modifying intervention available for the treatment of allergy [6, 7].

The most currently approved routes of AIT administration are subcutaneous immunotherapy (SCIT), involving monthly injections, and sublingual immunotherapy (SLIT), involving a daily dosing. Both are given over a period of 3–5 years. Both routes consistently demonstrated efficacy in reducing allergic symptoms and symptomatic drug use [8]. SCIT requires monthly doctors’ visits, but not daily dosing. SLIT can be self-administered at home following the initial dose, and is generally considered to have a better safety profile than SCIT [9, 10]. A key difference between the two routes of administration is that SLIT requires (on average) at least 50–100 times more allergen than SCIT to elicit a similar level of efficacy [9] and consequently, low-dose SLIT is generally ineffective [11].

Sublingual immunotherapy delivery systems include tablets and aqueous or glycerinated liquid allergen extracts (‘SLIT drops’) [9]. In the former system, a rapidly dissolving tablet is placed under the tongue and the contents then swallowed once dissolved, while in the latter, an aqueous allergen extract is administered as drops, which are held under the tongue for a few minutes and then swallowed, or in some cases, spat out, but this latter modality has been progressively abandoned [9].

The SLIT maintenance dose, typically corresponds to the pre-specified maximum treatment dose or the maximum tolerated dose for any patient with respiratory allergies [12], and there is a relationship between the maintenance dose of allergen, the clinical efficacy, the administration regimen, and the adherence [13].

A convincing body of evidence has shown that SLIT involves a dose–response relationship [14], and it is important to use a proven clinically effective dose. Studies using 5-grass pollen or house dust mite (HDM) tablets [15, 16] or grass pollen, birch pollen or HDM drops [17–19] for the treatment of respiratory allergies have confirmed that the daily 300 index of reactivity (IR) dose offers optimal efficacy and tolerability.

Here, we review the factors to consider when selecting the optimal dose of SLIT with natural allergens, and the rationale and supporting evidence for the use of the 300 IR dose for SLIT tablets and drops.

## Choosing the optimal dose of SLIT: what factors should be considered?

### How is allergen extract potency measured?

There is considerable variability in how allergen extract potency is measured and reported worldwide [8, 20], and manufacturers use a variety of different units of measurement [10]. This makes it difficult to compare studies. In Europe, allergen extract potency is reported in units based on an in-house reference, with some European product manufacturers using reference standards based on titrated skin testing of allergic patients. Biological potency can vary between different allergen preparations that are rated as having the same allergenicity.

**Fig. 1:**
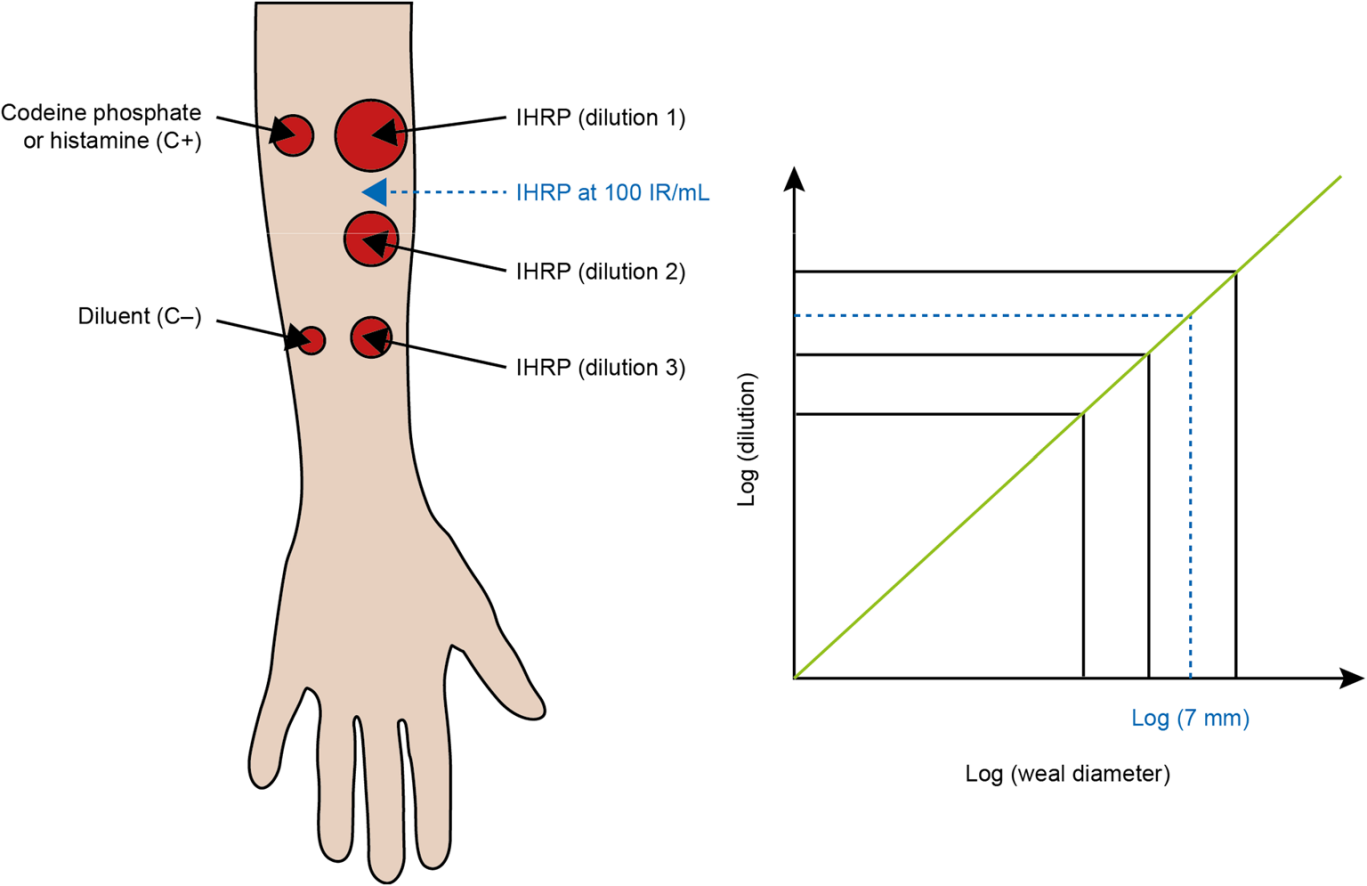
Defining a standardized dose using the index of reactivity. An allergen extract termed the IHRP is said to have a concentration of 100 IR/mL when, during skin prick-testing using a Stallerpoint^®^ needle on 30 subjects who are sensitized to the corresponding allergen source, it triggers a wheal size of 7 mm (geometric mean). Codeine phosphate or histamine serve as positive controls (C+), and diluent only is used as a negative control (C−) and to assess any background effects unrelated to the allergen extract. *IHRP* in-house reference preparation; IR, index of reactivity

Two SLIT therapies are currently licensed in both the EU and the US: Oralair^®^ (OA) (Stallergenes, Antony, France) and Grazax™/Grastek^®^ (GRA) (ALK-Abello, Hørsholm, Denmark/Merck, Kenilworth, NJ, USA). Stallergenes applies IR to assess allergenicity, in which an allergen extract contains 100 IR/mL when it induces a wheal diameter of 7 mm in 30 patients sensitized to this allergen (geometric mean) on a skin prick-test [21]. All Stallergenes SLIT drop (Staloral^®^), tablet, and diagnostics preparations are standardized in IR (Fig. 1) and produced using the same active pharmaceutical ingredients, and the same allergen source and technique. ALK-Abellò uses IR as a measure of allergenicity (formerly Allerbio SA, Varennes-en-Argonne, France) for its SLIT drop products (Osiris^®^ marketed in France and also known as SLIT One^®^ ULTRA in other countries but expressed in SRU, Standard Reactivity Unit), but the wheal diameter corresponding to 100 IR is 6 mm, instead of 7 mm [20]. ALK-Abello/Merck also uses the standardized quality tablet unit (SQ-T) as a measure of allergenicity. GRA is a 75,000 SQ-T oral lyophilizate tablet for SLIT containing a 1-grass-pollen allergen extract from timothy grass (*Phleum pratense*).

There have been attempts to standardize measures of allergen extract potency. In the development of SLIT for grass pollen allergy, the bioequivalent allergy unit (BAU) is a unit of standardization used for allergen extracts. This is recognized and performed by the US Food and Drug Administration, and is based on the reaction to an intradermal test in highly allergic patients [20]. Using the BAU, OA (EXPAND) has over three-times more allergenicity than GRA (9000 vs 2800 BAU, respectively). Based on the manufacturers’ in-house assays, the major allergen content is 25 μg for OA and 15 μg for GRA. Limited information (such as assay type, reference materials, antibodies and protocols) is publicly available. A comparison of the potency of different AIT products using these last figures is not feasible [14].

### Administration regimens for AIT

AIT regimens can be classified as continuous (i.e. year-round) or discontinuous. These latter may be preseasonal only, coseasonal only, or precoseasonal. Precoseasonal discontinuous regimens are typically used for SLIT and have efficacy and safety comparable to perennial (year-round) regimens, while also offering economic benefits and improved adherence [13, 22, 23].

There are likely relationships between the administration regimen, the maintenance dose of allergen, and clinical efficacy [13]. A sufficient cumulative dose of allergen may also be important, but is it the daily unit dose or the cumulative dose which matters the most? The EMA guideline on the clinical development of products for AIT for the treatment of allergic diseases requires studies to be performed to establish a dose–response, after establishing a tolerated dose range [25].

A review of the available data on dose–response relationships was conducted in 2011 by the Task Force of the European Academy of Allergy and Clinical Immunology Immunotherapy Interest Group [14]. Fifteen dose-ranging studies fulfilled their criteria for inclusion; 12 reported a dose–response relationship for clinical efficacy, and several studies also reported a dose–response relationship for immunological and safety endpoints. However, due to the use of different reference materials and methodologies for the determination of allergen content, variations in study design, and choice of endpoints, no comparisons could be made between these studies [14].

The approved titration of OA varies with country and/or patient age, but typically involves a 3-day dose-escalation from 100 to 300 IR per day, after which a 300 IR tablet is used daily as the maintenance dose until the end of the pollen season [26], which is adequate to ensure clinical efficacy.

### Need for better quality and standardization of allergen products

Sublingual immunotherapy products should meet the following requirements: be composed of high-quality allergen materials; have a standardized quantity of allergen content, and provide clinical efficacy and safety [24]. Standardization is used to account for the natural variability that is evident in the biological source materials used to make SLIT products and the large differences that can exist in the protein composition and allergen content of the marketed products [10, 14], because these variations may affect the quality, potency and extent of immunomodulation of the SLIT drug [27]. Standardization of the dosage is also crucial, as low doses are ineffective and very high doses may lead to adverse reactions [28].

Sublingual immunotherapy potency may be decreased by storage temperature, contamination (which accelerates degradation of the extract), the effects of dilution when extracts are mixed (loss of potency is proportional to the extract’s dilution), type of allergen, diluents used, and preservatives added [28].

Standardization of allergen products is achieved by adjusting the potency (total allergenic activity) of the source material during manufacturing of the allergen product, to ensure batch-to-batch reproducibility [29, 30].

It has been suggested that mixing multiple allergens in a single vaccine may dilute the allergen’s final content and lead to a suboptimal therapeutic effect, because clinical efficacy is dose-dependent [28]. However, studies investigating this are lacking.

### Choice of low-dose versus high-dose SLIT

Sublingual immunotherapy requires at least 50–100 times more allergen than SCIT to achieve a similar level of clinical efficacy. An analysis of dose–response studies in AR has been conducted by the European Academy of Allergy and Clinical Immunology Immunotherapy Interest Group task force on dose effect. This found that, for grass pollen, low doses of allergen (e.g. 5–7 µg Phl p 5 per day) are ineffective, and daily doses of 15–25 µg of the major allergen protein have been demonstrated for significant clinical improvement, as measured by symptom scores with a different administration protocol [11].

The efficacy of SLIT is dose-dependent [10, 14, 24], as shown by both clinical and immunological endpoints in well-designed, adequately powered, randomized controlled trials [14]. This again highlights the importance of administering a clinically effective dose from the onset of treatment.

## Efficacy of 300 IR SLIT

A number of well-designed randomized controlled trials have consistently established 300 IR SLIT once-daily as the optimally safe and effective dose for AR due to grass pollen, HDM and birch pollen, and HDM-induced moderate, persistent allergic asthma (Table 1).

**Table 1:** Summary of the described 300 IR SLIT studies

Study	Design and objectives	Allergen preparation	Population	Treatment and enrolled patients	Endpoints	Main results
SLIT tablets						
*5-grass pollen tablet*						
Didier et al. [16]	Multinational, randomised, DBPC study Efficacy and safety	5-grass pollen extract (orchard, meadow, perennial rye, sweet vernal, and timothy grasses)	Age 18–45 years Men and women with moderate-to-severe grass pollen-related ARC for ≥2 years, with or without mild asthma	100 IR (n = 157) 300 IR (n = 155) 500 IR (n = 160) Placebo (n = 156)	RTSS^a^ Individual symptom scores (sneezing, runny nose, itchy nose, nasal congestion, watery eyes, itchy eyes) Symptom-free days during the pollen season Rescue medication use during the pollen season Quality of life (RQLQ) Patients’ global evaluation of treatment Safety	Both the 300 IR and 500 IR doses significantly reduced mean RTSS (3.58 ± 3.0, p = 0.0001; and 3.74 ± 3.1, p = 0.0006, respectively) vs placebo (4.93 ± 3.2) The score for the 100 IR group was not significantly different from placebo Analysis of all secondary efficacy variables (sneezing, runny nose, itchy nose, nasal congestion, watery eyes, itchy eyes, rescue medication usage, and quality of life) confirmed the efficacy of the 300 IR and 500 IR doses No serious side effects were reported
Larsen et al. [31]	Single-centre, randomised, DBPC study Safety	5-grass pollen allergens (orchard, meadow, perennial rye, sweet vernal, and timothy grasses)	Age 18–50 years Men and women with grass pollen-induced AR, with or without mild asthma	Groups 1 and 2: incremental 100 IR, 200 IR, 300 IR, 400 IR and 500 IR SLIT (n = 6) or placebo (n = 4) (Group 1 daily and Group 2 every second day) Groups 3 and 4: repeated constant 300 IR and 500 IR SLIT, respectively (Group 3: n = 6, Group 4: n = 5) or placebo (Group 3: n = 1, Group 4: n = 2)	AEs, focusing on date of onset, occurrence, duration, intensity, action taken, outcome, and relationship to study drug Definition and grading of allergic side effects according to WHO	300 IR SLIT (Group 3) administered in a constant dose and incremental doses up to 500 IR (Groups 1 and 2) was generally well tolerated The majority of AEs were mild to moderate Most common AEs: oral pruritus, throat irritation, swollen tongue Severe local AEs (swelling of throat) were observed only for Group 4 No serious systemic AEs were reported
Malling et al. [32]	Multinational, randomised, DBPC study Efficacy and safety	5-grass pollen extract (orchard, meadow, perennial rye, sweet vernal, and timothy grasses)	Age 18–45 years Men and women with moderate-to-severe grass pollen-induced ARC for at least 2 years, with or without mild asthma	For Group 1 (high specific IgE), Group 2 (high symptom scores), Group 3 (high skin sensitivity) and Group 4 (any of Groups 1, 2 or 3), respectively: ITT population (n = 569) 100 IR SLIT (n = 55, 61, 34, 105) 300 IR SLIT (n = 59, 47, 32, 100) 500 IR SLIT (n = 57, 60, 39, 103) Placebo (n = 80, 56, 37, 105) Safety population (n = 628) 100 IR SLIT (n = 62, 67, 37, 117) 300 IR SLIT (n = 69, 52, 36, 112) 500 IR SLIT (n = 66, 66, 44, 117) Placebo (n = 82, 59, 40, 112)	ARTSS^a^ Individual symptom scores RTSS at the peak of the pollen season Symptom-free days during the pollen season Rescue medication use during the pollen season Quality of life (RQLQ) Patients’ global evaluation of treatment Safety	Across the 4 groups, ARTSS (±SD) for 300 IR was 3.91 ± 3.16 (Group 1), 3.83 ± 3.14 (Group 2), 2.55 ± 2.13 (Group 3) and 3.61 ± 2.97 (Group 4) Group 1: ARTSS did not differ significantly with different doses of SLIT Groups 2, 3 and 4: 300 IR and 500 IR SLIT doses were significantly more effective than 100 IR and placebo (p ≤ 0.035) ARTSS was comparable in patients with or without grass-pollen asthma, as well as for mono- or polysensitised patients All doses of SLIT were considered safe in the patients investigated
Cox et al. [34]	Multicentre, randomised, DBPC, parallel-group study Efficacy and safety	5-grass pollen allergen extract (orchard, meadow, perennial rye, sweet vernal, timothy)	Age 18–65 years Men and women with documented grass pollen-related ARC for at least 2 previous grass pollen seasons, with or without mild asthma	300 IR (n = 210) Placebo (n = 228)	DCS^a^ Daily symptom scores Symptom-free days during the pollen season Rescue medication use during the pollen season Quality of life (RQLQ) Patients’ global evaluation of treatment Safety	Mean DCS over the pollen period was significantly lower in the 300 IR group vs the placebo group (LS mean difference: 20.13; 95 % CI: 20.19, 20.06; p = 0.0003; relative reduction: 28.2 %; 95 % CI: 13.0 %, 43.4 %) 300 IR 5-grass pollen SLIT was well tolerated Most frequently reported AEs: oral pruritus, throat irritation, nasopharyngitis There were no reports of anaphylaxis
Wahn et al. [35]	Multinational, randomised, DBPC study	5-grass pollen allergen extract (orchard, meadow, perennial rye, sweet vernal, timothy)	Age 5–17 years Male and female children with grass pollen-related moderate-to-severe ARC for at least 2 years, with or without mild asthma	300 IR (n = 131) Placebo (n = 135)	RTSS^a^ Individual symptom scores Rescue medication intake Safety	RTSS for the 300 IR group was significantly improved by 28.0 % (median improvement 39.3 %) vs placebo (p = 0.001) Rescue medication use and proportion of days using rescue medication during the pollen season were both significantly reduced in the 300 IR group vs placebo (p = 0.0064 and p = 0.0146) AEs were generally mild or moderate and in keeping with the known safety profile of SLIT, and no serious side effects were reported
Didier et al. [36]	Multicentre, randomised, DBPC, parallel-group study Efficacy and safety	5-grass pollen allergen extract (orchard, meadow, perennial rye, sweet vernal, timothy)	Age 18–50 years Men and women with documented grass pollen-related ARC for ≥2 previous grass pollen seasons, with or without mild asthma	300 IR (2 M, administered 2 months prior to start of pollen season) (n = 117) 300 IR (4 M, administered 4 months prior to start of pollen season) (n = 127) Placebo (n = 133)	DCS^a^ Individual symptom scores ACS Symptom-free days during the pollen season Rescue medication use during the pollen season Safety	Patients were treated for 3 consecutive years and followed up for 2 years post-treatment During the first post-treatment year, a statistically significant decrease vs placebo in LS mean DCS was noted in patients previously receiving active treatment [300 IR (2 M): −31.1 %, p = 0.0019, 300 IR (4 M): −25.3 %, p = 0.0103) During the second post-treatment year, patients in the 300 IR (4 M) group, but not the 300 IR (2 M) group, showed a statistically significant decrease in LS mean DCS vs placebo (−28.1 %, p = 0.0478) The significant efficacy in the post-treatment years compared favourably with that during the 3 prior years of active treatment A statistically significant difference vs placebo was also noted in secondary efficacy measures in both post-treatment years (except for daily RTSS in year 5) In the absence of any active treatment, the safety profile was similar in the active vs placebo group during either post-treatment year
Horak et al. [37]	Single-centre, randomised, DBPC, parallel-group study	5-grass pollen allergen extract (orchard, meadow, perennial rye, sweet vernal, timothy)	Age 18–50 years Men and women with moderate-to-severe seasonal grass pollen-related ARC for ≥2 previous pollen seasons, with or without mild asthma	300 IR (n = 45) Placebo (n = 44)	ARTSS^a^ Nasal airflow, nasal secretion weight and cutaneous reactivity Safety	A significant treatment effect was achieved after the first (p = 0.0042) and second months (p = 0.0203), which was maintained to the fourth month (p = 0.0007) In the 300 IR group, mean ARTSS (±SD) decreased at each challenge (week 1: 7.40 ± 2.68, month 1: 5.89 ± 2.43, month 2: 5.09 ± 2.09, month 4: 4.85 ± 2.00) Mean ARTSS improved by 29.3 % (median: 33.3 %) vs placebo Most frequent local AEs: oral pruritus, ear pruritus, throat irritation
Antolin et al. [48]	Multicenter, observational, cross sectional study	5-grass pollen allergen extract (orchard, meadow, perennial rye, sweet vernal, timothy)	Age ≥6 years Patients with moderat to severe ARC to grass pollen	591 patients	Patients’ HRQoL Treatment satisfaction	Good level of satisfaction, with an average score of 69.2, on a 0–100 scale (100 being highest satisfaction). HRQoL questionnaires results were also favourable, mean (SD) scores were 1.40 (1.1) in adults, 1.33 (1.1) in adolescents and 1.15 (1.1) in children, with average scores much closer to 0 (no impairment) than to 6 (severe impairment)
*HDM tablet*						
Bergmann et al. 2014 [15]	Randomised DBPC study Efficacy and safety	HDM standardised extract	Age 18–50 years Adults with moderate-to-severe HDM-associated AR for ≥1 year	500 IR (n = 169) 300 IR (n = 170) Placebo (n = 170)	AAdSS^a^ Individual symptom score Rescue medication use Onset of action Patients’ global evaluation of treatment Safety	Both the 500 IR and 300 tablets significantly reduced mean AAdSS vs placebo by −20.2 % (p = 0.0066) and −17.9 % (p = 0.0150), respectively Efficacy of both doses was maintained during the treatment-free follow-up phase Onset of action was at 4 months Participants’ global evaluation of treatment success was significantly higher in the 500 IR and 300 IR groups vs placebo (p = 0.0206 and p = 0.0001, respectively) AEs were generally application-site reactions, and there were no reports of anaphylaxis
Okamoto et al. [38]	Multicenter randomized, DBPC, parallel-group design Efficacy and safety	HDM standardised extract	Age 12–64 years Adults with moderate-to-severe HDM-associated AR for ≥2 year	500 IR (n = 296) 300 IR (n = 315) Placebo (n = 316)	AAdSS^a^ Physicians’ intranasal examination Quality of life Patients’ global evaluation of treatment Safety	Both the 500 IR and 300 tablets significantly reduced mean AAdSS vs placebo by −13.12 % (p < 0.001) and −18.2 % (p < 0.001), respectively Both nasal mucosal swelling and rhinorrhea were markedly and significantly improved in both active treatment groups. In the Japanese RQLQall 3 domains showed a statistically significant improvement difference in the 300 IR group compared to placebo. Participants’ global evaluation of treatment success was significantly higher in the 500 IR and 300 IR groups vs placebo (p = 0.0002 and p < 0.0001, respectively) AEs were generally application-site reactions, and there were no reports of anaphylaxis
Ferrés et al. [39]	Retrospective, observational, single-centre study Efficacy and safety	HDM standardised extract	Age 6–18 years Patients with AR who were mono-sensitised to HDM, with or without mild asthma	300 IR (n = 78)	Global assessment of SLIT efficacy measured using a VAS^a^ Rhinitis medication consumption score Global asthma score Safety	Significant improvement in allergy severity at 6 months vs baseline (7.3 ± 4.6 vs 4.0 ± 1.7 cm on the VAS, respectively; p < 0.001), which was maintained throughout the 4-year follow-up period Symptomatic medication use was significantly reduced in the first 6 months vs baseline (0.8 ± 1.6 points vs 4.6 ± 2.5 points, respectively; p < 0.001) and remained very low until the end of follow-up SLIT was well tolerated, and no anaphylactic or life-threatening reactions were reported
SLIT drops						
*5-grass pollen drops*						
Ott et al. [17]	Randomised, DBPC, parallel-group, multicentre study Efficacy and safety	5-grass pollen extract (orchard, meadow, perennial rye, sweet vernal, and timothy grasses)	Age 7.9–64.7 years Men and women with grass pollen-related ARC using symptomatic medication during the grass pollen season, with or without mild asthma	300 IR (n = 99) Placebo (n = 46)	Combined symptom and rescue medication score^a^ Individual symptom scores Safety	Median combined scores decreased by 44.7 % in the SLIT group and 14.7 % in the placebo group vs baseline, by the third pollen season (p = 0.0019) Similar changes were observed for symptom scores, with a successive decrease of 39.7 % (SLIT) and fluctuations between +13.6 and −1.5 % for placebo (p < 0.05) over the 3 pollen seasons Combined score (p = 0.0508) and symptom score improvements (p = 0.0144) with SLIT continued during follow-up SLIT was well tolerated, and no serious systemic or anaphylactic reactions were reported
*Birch pollen drops*						
Worm et al. [19]	Randomized, DBPC, multicentre study Efficacy and safety	Birch pollen allergen standardised extract	Age 18–65 years Men and women with birch pollen-related ARC for ≥2 previous birch pollen seasons that required intake of symptomatic treatments, with or without OAS, with or without mild asthma	300 IR (n = 283) Placebo (n = 289)	AAdSS over the second pollen season^a^ AAdSS over the first pollen season Individual symptom scores Rescue medication use during the pollen season Quality of life (RQLQ) Safety	LS mean AAdSS was significantly lower in the 300 IR group than in the placebo group (LS mean difference -2.04, 95 % CI [−2.69, −1.40], (p < 0.0001) over the second pollen season (relative reduction of 30.6 %) Improvements in AAdSS were similar in patients with and without OAS (relative LS mean differences of −33.6 and −28.4 %, respectively) A significant reduction in LS mean AAdSS was also observed over the first pollen season SLIT was well tolerated, and no anaphylaxis was reported Most frequently reported AEs: application-site reactions (oral pruritus), throat irritation, mouth edema
*Grass and tree pollen drops*						
Seidenberg et al. 2009 [44]	Large observational study Safety	5-grass pollen (cocksfoot, meadow, rye, sweet vernal, and timothy grasses) or tree pollen (birch, alder, and hazel) allergen standardised extract	Age 5–17 years Children and adolescents with AR for ≥1 year due to grass or tree pollen, with or without mild to moderate asthma	Ultrarush titration high-dose 300 IR SLIT (n = 193)	Frequency and intensity (mild, moderate, severe) of expected local, gastrointestinal, and generalised AEs^a^ Patients’ and investigators’ global assessment of tolerability	Ultrarush titration was well tolerated During ultrarush titration, 60 patients (31 %) reported 117 predominantly mild and local AEs, which resolved within 150 min During the maintenance phase, 562 AEs were reported Most frequently reported local events: oral pruritus, burning sensation, lip or tongue swelling, gastrointestinal symptoms Most frequently reported systemic events: rhinoconjunctivitis, asthma There was 1 clinically significant asthma event in an 11-year old boy with known asthma, who resumed SLIT after 4 days
*HDM drops*						
Wang et al. [18]	DBPC, randomised study Efficacy and safety	HDM standardised extract	Age 14–50 years Patients with mild or moderate, persistent, HDM-induced asthma for ≥1 year	300 IR (n = 308) Placebo (n = 157)	Well-controlled asthma for ≥16 of the last 20 weeks of treatment^a^ Totally controlled asthma Safety	Well-controlled asthma was achieved by 85.4 and 81.5 % of patients in the 300 IR and placebo groups, respectively (p = 0.244) Post hoc analysis by asthma severity found significant clinical benefits in actively treated subjects with moderate asthma at baseline (n = 175), but not those with mild asthma: Greater achievement of well-controlled asthma (300 IR: 80.5 %, placebo: 66.1 %; p = 0.021) and totally controlled asthma (300 IR: 54.0 %, placebo: 33.9 %; p = 0.008) Higher percentage of patients with an asthma control questionnaire score <0.75 (300 IR: 56.6 %, placebo: 40.0 %; p = 0.039) Greater mean reduction in inhaled corticosteroid use (300 IR: 218.5 μg, placebo: 126.2 μg; p = 0.004) 300 IR was well tolerated, and no treatment-related serious AEs were reported
Trebuchon et al. [46, 47]	Retropsective, multicenter, observational study	HDM standardised extract	Age ≥5 years Children and adults with respiratory allergy and proven sensitization to HDM	1289 patients	Physician perception of patient satisfaction Compliance with treatment	Over 84 % of adult and pediatric patients with AR (with or without asthma) were satisfied and adhered to their treatment
*Grass and tree pollen, and HDM drops*						
Corzo et al. 2012 [45]	Multicentre, observational, epidemiological study Safety	5-grass pollen, olive tree pollen or HDM standardised extracts	Age 5–15 years Children and adolescents with AR, ARC and/or bronchial asthma due to grass pollen, olive pollen and/or HDM	300 IR (n = 74)	Tolerability and occurrence of local oral, systemic and gastrointestinal AEs^a^	After the first administration, 22 % of children had self-limiting pruritus, 4 % had moderate OAS (intense oropharyngeal itching) and 1 patient had diffuse abdominal pain No systemic reactions were recorded No patients reported problems during the maintenance phase, and there were no discontinuations of therapy

*AAdSS* Average Adjusted Symptom Score, *ACS* Average Combined Score, *AE* adverse event, *AR* allergic rhinitis, *ARC* allergic rhinoconjunctivitis, *ARTSS* Average Rhinoconjunctivitis Total Symptom Score, *CI* confidence interval, *DBPC* double-blind, placebo-controlled, *DCS* Daily Combined Score, *HDM* house dust mite, *IR* index of reactivity, *LS* least squares, *OAS* oral allergy syndrome, *RQLQ* Rhinoconjunctivitis Quality of Life Questionnaire, *RTSS* Rhinoconjunctivitis Total Symptom Score, *SD* standard deviation, *SLIT* sublingual immunotherapy, *VAS* visual analogue scale, *WHO* World Health Organization

^a^Denotes primary outcome measure

### SLIT tablets

#### Five-grass pollen tablet

The appropriateness and effectiveness of the 300 IR dose for the treatment of grass pollen-associated allergy has been extensively characterized. In a randomized, double-blind, placebo-controlled (DBPC), dose-ranging study, adults with grass pollen-induced allergic rhinoconjunctivitis (ARC) received either 100 IR, 300 IR or 500 IR doses of 5-grass pollen tablet or placebo, initiated 4 months before the estimated pollen season and continued throughout the season [16]. The 100 IR dose was not significantly different from placebo. In contrast, the 300 IR dose was effective from the first pollen season [16], and it provided excellent efficacy with a favorable risk–benefit profile [15, 16, 31, 32]. A dose response was not demonstrated between the 300 IR and 500 IR SLIT tablet formulations, and they showed comparable efficacy during the peak pollen season [16, 33].

The short-term efficacy of the 300 IR dose, initiated either 2 or 4 months before the estimated pollen season and continued throughout the season, was subsequently confirmed in two studies in adults [34] and children [35], respectively. In the former, adults achieved a significant reduction in least squares mean Daily Combined Score (DCS) with 300 IR 5-grass pollen tablet treatment, compared with placebo (p < 0.001), irrespective of sensitization status or the presence of mild comorbid asthma [34]. In the latter, children and adolescents with confirmed grass pollen-associated ARC for at least 2 years (N = 278) had a significantly decreased Average Rhinoconjunctivitis Total Symptom Score (ARTSS) after 300 IR 5-grass pollen tablet treatment, compared with placebo [35]. Again, the presence of comorbid mild asthma or sensitization status had no significant effect on the efficacy findings [35].

The long-term efficacy of the 300 IR dose, administered discontinuously as a pre- and coseasonal treatment (with a treatment-free period for the other months of the year, starting after the season ends), has also been demonstrated. In a randomized, DBPC, parallel-group field study in patients with grass pollen-induced ARC, a statistically significant difference in mean DCS versus placebo was observed in patients using the 300 IR 5-grass pollen tablet over 3 years of treatment, beginning 4 months prior to the estimated start of the grass pollen season until season’s end. This treatment effect was prolonged for up to 2 years post-treatment [36].

The 300 IR dose shows a rapid onset of action. In a randomized, DBPC, parallel-group allergen-challenge chamber study in patients with grass pollen-induced ARC, after 1 week of treatment, a difference in ARTSS was evident between 300 IR 5-grass pollen tablet and placebo. After 1 month of treatment, this improvement was significantly higher for the 300 IR dose versus placebo and was maintained over the following months [37].

Taken together, these studies in patients with grass pollen-induced ARC demonstrate that the 300 IR dose has a rapid onset of action and is effective in both adults and children over the short term, and when administered discontinuously over the long term, and is associated with a prolonged benefit, even after cessation of treatment.

#### House dust mite tablet

Two randomized, DBPC, pivotal studies have demonstrated the efficacy of the 300 IR SLIT tablet for HDM-induced AR.

The first study was conducted over 2 years in adults with confirmed HDM-associated AR (with or without intermittent asthma) who received either a 300 IR or 500 IR dose of HDM tablet or placebo daily for 12 months, and were then followed up during the subsequent treatment-free year [15]. In the first year, patients in the 300 IR group experienced a significant reduction in mean Average Adjusted Symptom Score (AAdSS), compared with those receiving placebo. This efficacy was maintained during the following treatment-free year, with these patients having significantly lower AAdSS versus placebo [15]. Patients who received the 300 IR dose also had a lower ARTSS in years 1 and 2, respectively, than those who received placebo. A significant overall improvement was demonstrated in the Rhinoconjunctivitis Quality of Life Questionnaire (RQLQ) score in the 300 IR group versus the placebo group in the first year, and at the end of treatment, the proportion of patients reporting marked improvement in symptoms was higher in the 300 IR group than in the placebo group, with greater treatment success [15].

The second, large-scale study was conducted in Japanese adults with confirmed HDM-associated AR (with or without intermittent asthma), who received either a 300 IR or 500 IR dose of HDM tablet or placebo daily for 12 months [38]. Patients receiving the 300 IR dose had a significant lower AAdSS, and a significant lower ARTSS, in the last 2 months of treatment, compared with their counterparts receiving placebo. This was accompanied by significant improvements in QoL, and by end of treatment, a marked improvement in symptoms, compared to the placebo group. In the subset of adolescents, those receiving the 300 IR dose also had a statistically significant lower AAdSS than those receiving placebo [38].

### SLIT drops

The 300 IR SLIT drop preparations (Staloral^®^) are indicated in IgE-mediated respiratory allergic diseases, mainly involving rhinitis, conjunctivitis, rhinoconjunctivitis or asthma (mild to moderate) of a seasonal or perennial nature.

#### Five-grass pollen drops

The material to produce tablets and drops is exactly the same. Like the 5-grass pollen tablet, a long-term effect has been shown for 5-grass pollen drops. A randomized, DBPC study in patients with grass pollen-induced AR evaluated the efficacy, carry-over effect and safety of coseasonal treatment with 5-grass pollen drops using ultra-rush titration with increasing doses up to 300 IR [17]. Treatment consisting of a daily intake of a 300 IR tablet throughout the season was effective from the first season onwards, with increasing efficacy observed over 3 years of treatment. There was a trend for a carry-over effect of seasonal SLIT (low pollen exposure in follow-up year) [17].

#### Birch pollen drops

A large, randomized, DBPC study in patients with birch pollen-induced ARC evaluated the efficacy and safety of treatment with 300 IR birch pollen drops, beginning 4 months before the estimated pollen season start and continuing until season’s end [19]. Pre- and coseasonal treatment with 300 IR birch pollen drops demonstrated sustained efficacy over 2 consecutive pollen seasons. Over the first and second birch pollen periods, a significant decrease in least squares means AAdSS versus placebo was noted in the 300 IR group. The least squares mean Average Rescue Medication Score (ARMS) versus placebo over the first and second pollen seasons was also significantly decreased with 300 IR birch pollen drops therapy [19]. This was accompanied by significant improvements in RQLQ score over both periods, respectively, compared with placebo [19].

#### House dust mite drops

A randomized, DBPC study investigated the efficacy and safety of daily 300 IR HDM drops over 12 months in Chinese adults with mild to moderate, persistent HDM-induced asthma [18]. The primary endpoint was well-controlled asthma (Global Initiative for Asthma classification) for ≥16 of the last 20 weeks of treatment, with total control of asthma being a secondary criterion. In a subgroup analysis, significant clinical benefits versus placebo were achieved with 300 IR HDM drops in patients with moderate, persistent asthma (>400–800 µg budesonide per day), but not mild asthma, at baseline. In the former group, there was a greater achievement of well-controlled asthma and totally controlled asthma, and a greater mean reduction in inhaled corticosteroid use [18]. A retrospective, observational study found that children with HDM-associated AR (N = 78) showed a significant improvement in asthma symptoms, and a reduction in asthma medication use, following 300 IR HDM tablet treatment [39].

## Tolerability and safety of 300 IR SLIT

Currently, 300 IR SLIT preparations contain natural allergens, but the limited contact with effector cells results in much reduced systemic reactions, compared with SCIT [28].

The incidence of adverse events (AEs) with SLIT does not appear to be dose-dependent, unlike SCIT, where increased frequencies of AEs are associated with higher allergen doses [14].

Safety has been evaluated in SLIT doses up to 1125 times greater than those used for SCIT [21]. Studies using 5-grass pollen or HDM tablets [15, 16] or grass pollen, birch pollen or HDM drops for the treatment of AR/ARC [17, 19] or HDM-induced mild to moderate, persistent asthma [18] have confirmed that the 300 IR per day dose offers optimal tolerability. The 300 IR dose of SLIT was well tolerated in children, adolescents and adults, even when administered as coseasonal treatment and even with ultra-rush titration schemes in SLIT drops [40]. The most frequently reported AEs were application-site reactions, and there was no report of anaphylactic shock. Rates of treatment-emergent AEs (TEAEs) were generally comparable between 300 IR SLIT and placebo for SLIT solutions and 5-grass pollen tablet, and serious AEs were rare [41, 42]; for the 300 IR HDM tablet, AE incidence was higher than in the placebo group. In clinical trials of the 5-grass pollen tablet, at least one TEAE was reported in 76.9 % of patients receiving active treatment and in 69.8 % of placebo-treated patients [41]. AEs are generally mild or moderate in severity, and rarely lead to treatment discontinuation. Furthermore, AEs tend to decline in frequency and severity over time and with repeated treatment [41]. In a study in 94 adult asthmatics exposed to doses of HDM tablet up to 2000 IR, there were no reports of anaphylaxis nor use of epinephrine, and no serious AEs [43].

Long-term studies have shown that when administered over consecutive seasons, the frequency of AEs decreased over each consecutive year of treatment with 5-grass pollen tablets or drops [17, 40, 41].

A large observational study in children and adolescents with AR due to grass or tree (birch, alder, hazel) pollen for ≥1 year (with or without intermittent asthma) demonstrated the favorable safety and tolerability of an ultra-rush, high-dose SLIT drops regimen reaching a maintenance dose of 300 IR within 90 min [44]. Treatment was initiated with a sublingual application of 30 IR SLIT, followed by 3 applications of increasing dosage at 30-min intervals (30-90-150-300 IR regimen). The subsequent maintenance phase at 300 IR (or highest tolerated dose) lasted up to 4 months, depending on length of pollen season. During ultra-rush titration, predominantly mild and local AEs occurred, which resolved within 150 min. During the maintenance phase, of the AEs reported, the most frequent local events were oral pruritus, burning sensation, lip or tongue swelling, and gastrointestinal symptoms, and the most frequent systemic events were rhinoconjunctivitis and asthma. A single clinically significant asthma event occurred in a boy aged 11 years with known asthma (dysphagia and dyspnea on day 4, immediately after the 300 IR dose). Symptoms resolved with prednisone, clemastine and theophylline treatment, and after 4 days, SLIT was resumed with gradually increasing doses to 300 IR [44].

In a Spanish study, SLIT drops have also demonstrated a favorable safety profile when administered by non-conventional, ultra-rush dosing regimens using SLIT extracts standardized in IR/mL (Staloral 300 Rapid^®^). This prospective, observational, multicenter study evaluated the tolerability of SLIT drops administered as an ultra-rush 300 IR dose to children with a respiratory allergic disease (AR, ARC and/or bronchial asthma) induced by HDM, grasses or olive trees, but with no prior history of AIT [45]. Treatment was initiated with 2 sublingual applications of ultra-rush 300 IR SLIT, followed by 3 applications at 30-min intervals. The subsequent maintenance dosing regimen was 5 once-daily 300 IR sublingual applications. In this study, ultra-rush 300 IR SLIT appeared to be better tolerated than the conventional dosing regimen in this pediatric population, which may facilitate adherence and eliminate the need for dose escalation [45].

## Adherence and patient satisfaction

A large, retrospective, multicenter, observational study of HDM drops, in which patients were titrated to a maintenance dose of 300 IR daily, evaluated physician perception of patient satisfaction and compliance with treatment. It was shown that both adult and pediatric patients with AR (with or without asthma) were highly satisfied and adhered to their treatment [46, 47]. Similarly, satisfaction rates were high in a multicenter, observational, cross-sectional study carried out in Spain in patients with grass pollen-induced ARC who were previously naïve to 300 IR 5-grass pollen tablet therapy. A safe, shorter initial-treatment scheme was used, in which titration to the 300 IR daily dose was reached over 5 days. This shorter conventional scheme with reduced titration period may improve the patient’s adherence to treatment [48–50]. However, persistence on therapy after the first dose still seems to be low [51].

## Summary and discussion

AIT is the only disease-modifying intervention available for the treatment of allergy [6, 7]. For a successful AIT, the chosen product should be of high quality, with a proven, sustained, documented and validated effective dose. Studies of SLIT have focused on defining the optimal dose of major allergen, and the administration frequency, duration of treatment, and number of treatment seasons [13].

Low-dose SLIT is generally ineffective, whereas high-dose SLIT has been demonstrated to be clinically effective and safe. For grass pollen, daily doses of 15–25 µg of the major allergen protein are typically required for significant clinical improvement [11].

There is substantial evidence demonstrating a dose–response relationship in SLIT. Studies using SLIT tablets [15, 16] or drops [17–19] for the treatment of AR/ARC and/or moderate, persistent asthma have confirmed that the 300 IR per day dose offers optimal efficacy and tolerability. The 300 IR dose is also appropriate and provides effective treatment for a variety of different allergens, offering simplicity of dosing, which may not be the case for other allergen preparations that use alternative measures of potency, depending on the allergen. 300 IR SLIT shows a favorable efficacy profile, reducing allergy-related symptoms associated with HDM or pollen, and decreasing the use of symptomatic rescue medications and asthma medication, sensitization status and/or the presence of mild asthma. In studies of SLIT that used AEs as a clinical endpoint, dose-dependent increases in efficacy were not associated with an increased frequency of AEs, which possibly reflects the different sites of action within the immune system.

The improved safety profile of SLIT compared with SCIT is probably due to the fact that oral antigen-presenting cells exhibit a tolerogenic phenotype, which reduces the induction of pro-inflammatory immune responses that lead to systemic allergic reactions [9]. Most side effects with 300 IR SLIT are mild to moderate local reactions involving the oral or gastrointestinal mucosa. If not adequately managed, these can lead to treatment discontinuation. Effective management allows patients to reach the maintenance dose with no further reactions [52].

The conventional dosing scheme for 300 IR SLIT drops (Staloral^®^) consists of two phases: a titration phase with an escalation of the dose and a maintenance phase with a stable dose. In this conventional scheme, the titration phase lasts for up to 10 days, with a possible dose increase from 10 IR to 300 IR [42]. Results from an analysis of clinical data collected from 640 patients treated using an ultra-rush one-day SLIT titration phase, with a dose increase from 30 IR to 240 IR or 300 IR, were similar to those observed using a titration phase over 12 days. However, it is important to note that the ultra-rush regimen should only be used in a hospital setting, under the close supervision of the prescribing physician.

300 IR SLIT is associated with high rates of adherence and satisfaction, demonstrating that this dose is acceptable to patients. This is likely due to the favorable clinical efficacy and tolerability profile as well as the convenience of 300 IR SLIT, which allows at-home administration in adults, adolescents and children. Further research would be valuable to investigate adherence rates of patients using 300 IR SLIT over successive pollen seasons, and improvement in QoL. “Increasing the effectiveness of adherence interventions may have a far greater impact on the health of the population than any improvement in specific medical treatments” [53].

## Conclusions

Successful AIT requires the use of high-quality products with a proven effective, standardized dose and biological potency. The dose is a key success factor and should be clearly defined. A robust body of clinical evidence pertaining to different allergens for AR and/or HDM-induced moderate, persistent asthma has established the 300 IR per day dose as offering optimal efficacy and safety in different modes of administration (tablets and drops). The 300 IR per day dose has also been shown to promote patient adherence to SLIT therapy, compared with lower daily doses, and may improve treatment outcomes.

## Abbreviations

AAdSS: Average Adjusted Symptom Score
AE: adverse event
AIT: allergen immunotherapy
AR: allergic rhinitis
ARC: allergic rhinoconjunctivitis
ARMS: Average Rescue Medication Score
ARTSS: Average Rhinoconjunctivitis Total Symptom Score
BAU: bioequivalent allergy unit
DBPC: double-blind, placebo-controlled
DCS: Daily Combined Score
EMA: European Medicines Agency
GRA: Grazax(r)
HDM: House Dust Mite
IR: index of reactivity
OA: Oralair^®^
RQLQ: Rhinoconjunctivitis Quality of Life Questionnaire
SCIT: subcutaneous allergen immunotherapy
SLIT: sublingual allergen immunotherapy
SQ: standardized quality
TEAE: treatment-emergent adverse event
